# Broccoli to the Lab: Green-Synthesized N-CQDs for Ultrasensitive “Turn-On” Detection of Norfloxacin in Food

**DOI:** 10.3390/s25206284

**Published:** 2025-10-10

**Authors:** Zubair Akram, Anam Arshad, Sajida Noureen, Muhammad Mehdi, Ali Raza, Nan Wang, Feng Yu

**Affiliations:** 1 Key Laboratory for Green Processing of Chemical Engineering of Xinjiang Bingtuan, School of Chemistry and Chemical Engineering, Shihezi University, Shihezi 832003, China; zubair.a.sidhu@gmail.com (Z.A.); anamarshad9293@gmail.com (A.A.); razaali2021@stu.shzu.edu.cn (A.R.); 2 Materials Chemistry Laboratory, Institute of Chemistry, The Islamia University of Bahawalpur, Baghdad-ul-Jadeed Campus, Bahawalpur 63100, Pakistan; sajida.noureen@iub.edu.pk; 3 College of Chemistry & Pharmacy, Northwest A&F University, Yangling 712100, China; kh.mehdiali@nwafu.edu.cn; 4 Carbon Neutralization and Environmental Catalytic Technology Laboratory, Bingtuan Industrial Technology Research Institute, Shihezi University, Shihezi 832003, China

**Keywords:** broccoli-derived carbon quantum dots, norfloxacin detection, fluorescence sensor, turn-on sensing, food safety

## Abstract

**Highlights:**

**What are the main findings?**

- A novel broccoli-derived nitrogen-doped carbon quantum dots (N-CQDs) was successfully synthesized via a green hydrothermal method, using 4-dimethylaminopyridine (DMAP) as both a nitrogen dopant and functionalizing agent; the N-CQDs have an average diameter of ~4.2 nm and emit bright blue fluorescence (λ_em_ = 445 nm under λ_ex_ = 360 nm).
- The N-CQDs-based sensor achieves a “Turn-ON” fluorescent response to norfloxacin (NFX) with a low detection limit of 0.30 nM (driven by hydrogen bonding and π–π stacking interactions suppressing non-radiative decay), high selectivity against common interferents (other antibiotics, organic acids, biomolecules), and excellent stability under varying pH, high ionic strength, and prolonged irradiation.
- Practical validation shows the sensor enables reliable NFX detection in spiked broccoli extract and milk samples, with recovery rates of 98.2–100.1% and relative standard deviations < 2.0%.

**What is the implication of the main finding?**

- The broccoli-derived N-CQDs synthesis strategy realizes a sustainable, green alternative to traditional carbon quantum dot preparation, aligning with the demand for eco-friendly analytical materials.
- The developed fluorescent sensing platform provides a sensitive, selective, and stable tool for rapid NFX detection, offering significant application potential in food safety monitoring (e.g., dairy products, vegetables) and environmental residue analysis.

**Abstract:**

The widespread presence of antibiotic residues, particularly norfloxacin (NFX), in food products and the environment has raised concern, underscoring the need for sensitive and selective detection methods. In this study, a novel broccoli-derived nitrogen-doped carbon quantum dots (N-CQDs) was synthesized via a green hydrothermal approach, 4-dimethylaminopyridine (DMAP) as both a nitrogen dopant and a functionalizing agent. The synthesized N-CQDs exhibit an average diameter of approximately ~4.2 nm and emit bright blue fluorescence, with a maximum emission at 445 nm upon excitation at 360 nm. A “Turn-ON” response toward NFX was achieved with a detection limit of 0.30 nM, attributed to hydrogen bonding and π–π stacking interactions that suppressed non-radiative decay. Moreover, the sensor demonstrates high selectivity for NFX, effectively distinguishing it from common interfering substances, including other antibiotics, organic acids, and biomolecules. The N-CQDs also exhibit excellent stability under diverse conditions, such as varying pH levels, high ionic strength, and prolonged irradiation. Finally, the practical applicability of the developed sensor was validated by detecting NFX in spiked broccoli extract and milk samples, with recovery rates ranging from 98.2% to 100.1% and relative standard deviations of less than 2.0%. This work presents a sustainable and efficient N-CQD-based fluorescent sensing platform, offering significant potential for rapid and reliable detection of NFX in food safety and environmental monitoring.

## 1. Introduction

The extensive use of antibiotics, such as norfloxacin (NFX), a potent fluoroquinolone, in human and veterinary medicines as well as aquaculture, has raised serious concerns regarding their environmental providence and potential impacts on human health [1,2,3,4]. NFX exhibits broad-spectrum antibacterial activity against both Gram-positive and Gram-negative aerobic organisms [5]. However, due to its incomplete metabolism and environmental persistence, NFX contaminates various food products such as milk and vegetables, as well as water sources [6,7,8]. Even at low concentrations in the effluents of sewage treatment plants and surface waters worldwide, residues of NFX present significant risks. These include allergic reactions, toxic impacts, and most critically the facilitation and dissemination of antimicrobial resistance (AMR) [9]. Compounding this threat, recent quantitative studies document alarming NFX contamination across environmental and food compartments: Surveys in South China reported NFX concentrations of 3.49–660.13 ng/L in water, 1.03–722.18 μg/kg in sediment, and 6.73–968.66 μg/kg in edible fish—highlighting not only environmental persistence but also bioaccumulation in food chains that expose humans. China’s annual veterinary use of ~400 tons of NFX further drives this contamination, as unmetabolized fractions of the antibiotic enter ecosystems via runoff and manure. Ecological risk assessments reinforce these concerns: sediment risk quotient values for NFX exceed 1, signaling high adverse risks to non-target organisms and aquatic food web disruption [10]. AMR has emerged as a major global health threat, reducing the effectiveness of existing antibiotic treatments and complicating the management of infectious diseases [11]. The strong link between antibiotic residues in the environment and the food chain, along with the escalating AMR crisis, underscores the urgent need for sensitive and reliable detection methods. Such methods are essential not only for residue monitoring but also for public health surveillance and AMR mitigation strategies.

Numerous analytical techniques have been developed to detect NFX, with liquid chromatography (LC) and electrochemical methods being the most common. LC, exceptionally high-performance liquid chromatography (HPLC), is widely regarded as the primary tool for NFX monitoring owing to its high selectivity and sensitivity [12]. Electrochemical sensors, particularly those modified with nanomaterials, also show potential for detecting pharmaceuticals in various matrices by enhancing sensitivity and selectivity [13,14,15]. Nevertheless, these traditional approaches have significant drawbacks. LC-based methods are expensive, time-consuming, and require sophisticated instrumentation and highly trained operators. Their complex sample preparation further renders them unsuitable for rapid on-site screening [16]. Although electrochemical sensors are fast and cost-effective, they are vulnerable to matrix interference and biofouling, which can weaken signals in real samples such as biofluids and environmental waters [17,18,19]. This gap highlights the need for alternative detection platforms that are simple, rapid, portable, and cost-effective yet maintain high sensitivity and selectivity for routine NFX monitoring.

In recent years, carbon quantum dots (CQDs), a class of zero-dimensional carbon nanomaterials with sizes typically smaller than 10 nm, have gathered significant attention [20,21]. These nanomaterials feature a unique sp^2^/sp^3^ hybridized carbon core and a surface rich in diverse functional groups. CQDs exhibit a remarkable set of properties that make them highly suitable for sensing applications [22]. These include strong and tunable photoluminescence (PL), high quantum yields (QY), excellent photostability and chemical stability, good water solubility, low cytotoxicity, and inherent biocompatibility [23,24]. Additionally, CQDs can be synthesized relatively easily from a wide range of carbon precursors, including abundant and inexpensive biomass materials, and their surfaces can be readily functionalized to tailor their properties for specific applications [24,25,26]. Their small size enables uniform dispersion and enhances interactions with target analytes, making them highly versatile for developing advanced sensor platforms.

The green synthesis of CQDs using biomass or waste materials as carbon sources has gained considerable interest due to its alignment with sustainability principles, cost-effectiveness, and the inherent potential to incorporate beneficial heteroatoms and surface functional groups [27]. Broccoli powder, derived from a common vegetable, represents a promising and largely unexplored biomass precursor. It is rich in phytochemicals that may produce the resulting CQDs with unique surface functionalities. N-doping is a widely adopted strategy to enhance the performance of CQDs. Introducing nitrogen atoms into the carbon lattice modulates the electronic structure of CQDs, significantly improving their PL QY and creating specific active sites on the CQD surface that interact with target analytes, thereby enhancing sensing selectivity and sensitivity [28]. In this study, 4-dimethylaminopyridine (DMAP) is used as a nitrogen source and surface functionalizing agent. DMAP, with its distinct pyridine ring and dimethylamino moiety, offers a unique chemical structure compared to more commonly used N-dopants such as urea or ammonia. Prior studies confirm its role as a nitrogen-containing precursor for synthesizing nitrogen-doped carbon dots (N-CDs), where it enhances photostability and fluorescence performance via intrinsic structural modulation. Nitrogen atoms from DMAP—particularly pyridinic species—modify CQD electronic structures by lowering unoccupied orbital energy levels, facilitating electron transfer, and reducing non-radiative decay. Unlike simpler dopants (e.g., urea), DMAP’s pyridine ring and amino groups introduce diverse nitrogen functionalities, enabling precise control over surface chemistry to tailor photoluminescent properties [29]. The synergistic combination of broccoli powder, which provides a complex carbon structure and innate oxygen functionalities, and DMAP, a specific nitrogen-containing organic molecule, is hypothesized to yield N-CQDs with distinctive surface chemistry. This tailored surface, rich in oxygen-containing groups from broccoli and specific nitrogen functionalities (e.g., pyridinic-N, amino-N) from DMAP, is expected to facilitate enhanced and selective interactions with NFX, potentially leading to promising complementary sensing characteristics compared to CQDs synthesized from simpler precursors or non-specific N-dopants. While biomass-derived CQDs have shown promise for antibiotic sensing, existing approaches often lack the specificity or sensitivity needed for targeted NFX detection, and none have leveraged the unique synergy of broccoli as a carbon precursor and DMAP as a dual nitrogen dopant/functionalizer. For example, Curcuma amada-derived carbon dots—among the more sensitive biomass-based probes—exhibit dual detection capabilities for tetracyclines and fluoroquinolones, but their LOD for fluoroquinolones (2 nM) remains nearly 7-fold higher than the 0.30 nM LOD of our broccoli-DMAP N-CQDs [30].

Fluorescence-based sensing combines high sensitivity with operational simplicity. Among various fluorescence sensing strategies, “turn-on” sensors, which exhibit an enhanced fluorescence signal upon analyte binding, are often preferred over “turn-off” (quenching) sensors [31]. “Turn-on” systems generally provide higher sensitivity because the increase in signal is measured against a low or dark background, reducing the likelihood of false-positive signals and improving the signal-to-noise ratio [32]. Several mechanisms can underlie a “turn-on” fluorescence response, including inhibition of photoinduced electron transfer (PET), disruption of Förster resonance energy transfer (FRET) to a quencher, aggregation-induced emission (AIE), or conformational changes in the fluorophore or sensor–analyte complex that block non-radiative decay pathways and enhance radiative emission.

The primary objective of this study is to address the critical need for simple, rapid, sensitive, and environmentally friendly methods for NFX detection, achieved by developing a novel “turn-on” fluorescent sensor. The sensor is based on N-CQDs synthesized via a facile, eco-friendly hydrothermal method, utilizing broccoli powder as the carbon source and DMAP as both the nitrogen source and surface functionalizing agent. The novelty of this work lies in several aspects: (i) the first use of the specific combination of broccoli powder and DMAP for the synthesis of N-CQDs targeted at antibiotic sensing; (ii) the development and characterization of a “turn-on” fluorescent sensing mechanism for NFX using these uniquely functionalized N-CQDs; and (iii) the demonstration of the sensor’s practical applicability for NFX detection in complex food matrices, especially broccoli extract, and milk. This research contributes to the growing field of sustainable nanomaterials for analytical applications, offering a promising tool for improving food safety and environmental monitoring.

## 2. Materials and Methods

### 2.1. Materials and Reagents

Broccoli powder was prepared from fresh broccoli florets purchased from a local market in Shihezi City, Xinjiang, China. The florets were thoroughly washed, dried overnight at 60 °C in an oven, and then finely ground with a grinder. 4-Dimethylaminopyridine (DMAP, ≥99%), norfloxacin (NOR, ≥98%), tetracycline, sulfamethoxazole (SMX, ≥98%), small biomolecules (urea, glucose, alanine, glycine), organic acids (ascorbic acid, citric acid, all analytical grade) and a model protein (bovine serum albumin, BSA) were purchased from Merck, China and used as received without further purification. Analytical grade ethanol was obtained from a local supplier. Deionized (DI) water with a resistivity of 18.2 MΩ·cm, produced by a Milli-Q system (Hyper PureX), was used throughout all experiments.

### 2.2. Synthesis of N-CQDs

N-CQDs were synthesized via a one-step hydrothermal method. In this typical synthesis, 1.5 g of broccoli powder and 0.1 g of DMAP were dispersed in 30 mL of DI water. The broccoli (1.5 g) and DMAP (0.1 g) ratio was optimized by testing three different mass ratios (1.5:0.05, 1.5:0.1, 1.5:0.2 g/g) and evaluating the key metrics: relative fluorescence enhancement (F/F_0_) of N-CQDs + 100 nM NFX. Lower DMAP concentrations (0.05 g) resulted in insufficient nitrogen doping, while higher DMAP concentrations (0.2 g) caused excessive surface functionalization (aggregation of N-CQDs). Thus, 1.5 g broccoli and 0.1 g DMAP were selected as optimal. The mixture was ultrasonicated for 30 min to ensure a homogeneous dispersion. Subsequently, the suspension was transferred into a 50 mL Teflon-lined stainless-steel autoclave and heated in an oven at 180 °C for 10 h. After the reaction, the autoclave was allowed to cool naturally to room temperature. The resulting dark brown solution was centrifuged at 10,000 rpm for 10 min to remove large particles and unreacted residues. The supernatant was filtered through a 0.22 µm microporous membrane. The filtered solution was dialyzed against DI water using a dialysis membrane Spectra/Por (MWCO 1000 Da) for 48 h, with the external water changed every 6 h, to remove small molecules and excess reactants. The purified N-CQD solution was stored at 4 °C for further use. A detailed schematic illustration of the N-CQDs synthesis process is provided in the Supplementary Materials (Figure S1). A portion of the solution was freeze-dried to obtain N-CQD powder for characterization.

### 2.3. Characterization Techniques

The morphology and microstructure of the synthesized N-CQDs were characterized using various analytical techniques. X-ray diffraction (XRD) patterns were recorded on a Bruker D8 Advance diffractometer in the 2θ range of 10° to 80°, with a step size of 0.02° and a scan speed of 2° min^−1^. Fourier transform infrared (FTIR) spectra were obtained using a (PerkinElmer-400 spectrum) spectrometer. X-ray Photoelectron Spectroscopy (XPS) measurements were performed on a Thermo K-Alpha XPS system equipped with a monochromatic Al Kα X-ray source. Survey scans and high-resolution scans for C1s, N1s, and O1s regions were acquired. Binding energies were calibrated using the C1s peak of adventitious carbon at 284.8 eV. Transmission electron microscopy (TEM) images were obtained using a JEOL JEM-F200 microscope operating at an accelerating voltage of 200 kV. Samples for TEM were prepared by drop-casting a diluted aqueous solution of N-CQDs onto a thin carbon-coated copper grid and allowing them to air-dry. UV-Visible Absorption spectra of the N-CQDs were recorded on a (UV-2600) spectrophotometer within a wavelength range of 200–800 nm, with ultrapure water serving as the blank reference. Fluorescence excitation and emission spectra were recorded using RF-6000 spectrofluorometer. The slit widths for excitation and emission were set to 3 nm. All measurements were conducted at room temperature (25 ± 2 °C) in ultrapure water.

### 2.4. Fluorescence Measurements for Norfloxacin Detection

A stock solution of N-CQDs (1 mg mL^−1^) was prepared in DI water. For sensing experiments, the N-CQD stock solution was diluted to an appropriate working concentration of 50 µg mL^−1^ in phosphate-buffer saline PBS, 0.01 M, pH 7.4. In this assay, 2 mL of the N-CQD working solution was placed in a quartz cuvette. Varying concentrations of NFX standard solution were added to the N-CQD solution in 5 µL increments, resulting in final NFX concentrations of 0, 2.5, 5, 10, 20, 50, 100, 150, 200, and 250 nM. The mixture was incubated for 5 min at room temperature to allow interaction before fluorescence measurements. Fluorescence emission spectra were recorded with an excitation wavelength of 360 nm, and the emission intensity at 445 nm was monitored. The fluorescence enhancement was quantified as F/F_0_, where F_0_ and F are the fluorescence intensities of the N-CQD solution at 445 nm in the absence and presence of NFX, respectively. Optimization of incubation time for N-CQD-NFX interaction. Relative fluorescence intensity (F/F_0_) of N-CQDs + 100 nM NFX was measured at 0, 2, 5, 10, 20, and 30 min. F/F_0_ reached a plateau at 5 min and remained stable for up to 30 min, indicating equilibrium of the interaction. Thus, 5 min was selected as the optimal incubation time.

### 2.5. Selectivity Studies

The selectivity of the N-CQD sensor for NFX was evaluated against a range of potential interfering substances. These included other antibiotics such as tetracycline and sulfamethoxazole (SMX), small biomolecules such as urea, glucose, alanine and glycine, organic acids (ascorbic acid and citric acid), and a model protein (bovine serum albumin, BSA). The concentration of NFX was typically set at 20 nM, while the concentration of interfering substances was 10-fold higher, 200 nM, to assess the sensor’s specificity. The fluorescence response (F/F_0_) was measured after the addition of each interfering substance alone to the N-CQD solution, and also after the addition of NFX to the N-CQD solution pre-mixed with the interferent.

### 2.6. Stability Studies

The effect of pH on the fluorescence intensity of the N-CQDs was investigated over a pH range of 1.0 to 14.0. The pH of the N-CQD solution was adjusted using 0.1 M HCl or 0.1 M NaOH solutions. Fluorescence intensity at 445 nm (excitation at 360 nm) was measured after equilibration at each pH value. The influence of ionic strength on the N-CQD fluorescence was studied by adding different concentrations of NaCl (ranging from 0 to 1.0 M) to the N-CQD solution. Fluorescence spectra were recorded after a 5 min incubation. The photostability of the N-CQDs was assessed by continuously putting the N-CQD solution under light. The N-CQDs exhibited excellent photostability under prolonged UV irradiation (365 nm, 10 W): after 120 min, they retained 92% of initial fluorescence intensity (Figure S3). This ensures reliable signal acquisition during fluorescence measurements. The fluorescence intensity at 445 nm was monitored at regular intervals.

### 2.7. Real Sample Preparation and Analysis

Fresh broccoli 5 g was homogenized with 20 mL of DI water. The mixture was centrifuged at 8000 rpm for 15 min, and the supernatant was filtered through a 0.22 µm syringe filter to obtain the broccoli extract. Commercially available pasteurized cow’s milk (10 mL) was deproteinized by adding 10 mL of acetonitrile, vortexing for 2 min, and then centrifuging at 10,000 rpm for 15 min. The clear supernatant was filtered through a 0.22 µm syringe filter.

The prepared broccoli extract and milk supernatant were then spiked with known concentrations of NFX (5, 10, and 20 nM). The spiked samples were analyzed using the developed N-CQD sensor as described in Section 2.4. The recovery (%) was calculated as (concentration detected/concentration spiked) × 100%. The relative standard deviation (RSD, %) was calculated from three replicate measurements.

## 3. Results and Discussions

### 3.1. Synthesis and Characterization of N-CQDs

**Figure 1:**
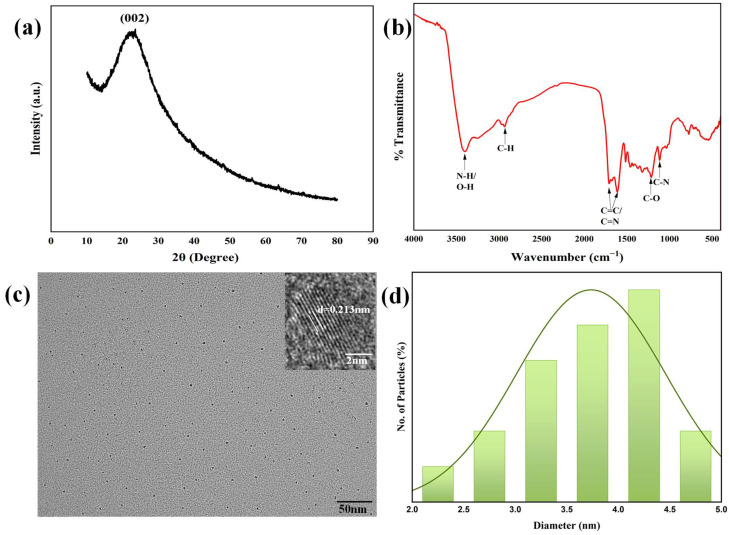
XRD pattern (**a**), FTIR spectrum (**b**), TEM and HR-TEM (inset) images (**c**), particle size distribution (**d**) of N-CQDs.

Firstly, the N-CQDs were successfully synthesized via a one-step hydrothermal method using broccoli powder and DMAP as precursors. The prepared N-CQDs were characterized by multiple techniques to elucidate their physicochemical properties. The morphological features and crystalline architecture of the as-synthesized N-CQDs were systematically interrogated using XRD, FTIR, TEM, HR-TEM, and XPS to validate their structural integrity and compositional homogeneity. The XRD analysis (Figure 1a) provides additional evidence for the graphitic structure, with a broad diffraction peak centered at 2θ ≈ 23°. This peak is attributed to the (002) reflection of graphitic carbon, arising from the periodic stacking of sp^2^-hybridized carbon layers [33]. The broadness of the peak suggests a combination of nanocrystalline domains and short-range structural order, typical of quantum dots with small particle sizes. Notably, the XRD results are in excellent agreement with the HR-TEM observations, reinforcing the formation of a graphitic core in the N-CQDs.

FTIR spectroscopy was employed to identify surface-bound functional groups, with the spectrum (Figure 1b) revealing a complex array of vibrational modes consistent with multifunctional surface chemistry. A prominent broad absorption band spanning 3200–3500 cm^−1^ dominates the high-wavenumber region, arising from the overlapping stretching vibrations of O-H (hydroxyl) and N-H (amine) groups, indicative of hydrophilic surface functionalities that enhance aqueous dispersibility [34]. A series of weaker yet distinct peaks centered at ~2900 cm^−1^ correspond to C-H stretching vibrations in aliphatic moieties, suggesting residual alkyl chains or surface-bound organic fragments from the synthesis precursor. A strong, well-defined absorption at ~1650 cm^−1^ emerges as a key spectral feature, attributable to the superimposed contributions of aromatic C=C stretching (consistent with graphitic core formation) and C=N vibrations, direct evidence of nitrogen incorporation into the carbon framework [35]. Additional diagnostic peaks at ~1250 cm^−1^ (C-O stretching) and ~1100 cm^−1^ (C-N stretching) further corroborate the presence of oxygen-containing functionalities (e.g., hydroxyls) and covalent nitrogen–carbon bonding, respectively, validating successful heteroatom doping and surface functionalization.

Representative TEM micrographs (Figure 1c) demonstrate that the N-CQDs exhibit a monodisperse spherical morphology with negligible aggregation, suggesting controlled nucleation and growth during synthesis. Particle sizes were measured using ImageJ 1.54g software by analyzing over 100 individual particles from three independent TEM images. The size distribution histogram, derived from measurements of over 100 particles (Figure 1d), reveals a narrow size distribution with a mean diameter of 4.2 ± 0.3 nm, consistent with previously reported values for high-quality N-CQDs [36]. The HR-TEM image (inset, Figure 1c) further elucidates the crystalline nature of the N-CQDs, displaying well-resolved lattice fringes with an interplanar d-spacing of 0.213 nm. This d-spacing corresponds precisely to the (100) lattice plane of graphitic carbon, confirming the presence of a hexagonal crystalline graphitic core, a critical structural feature for enhancing electron mobility and photophysical properties.

**Figure 2:**
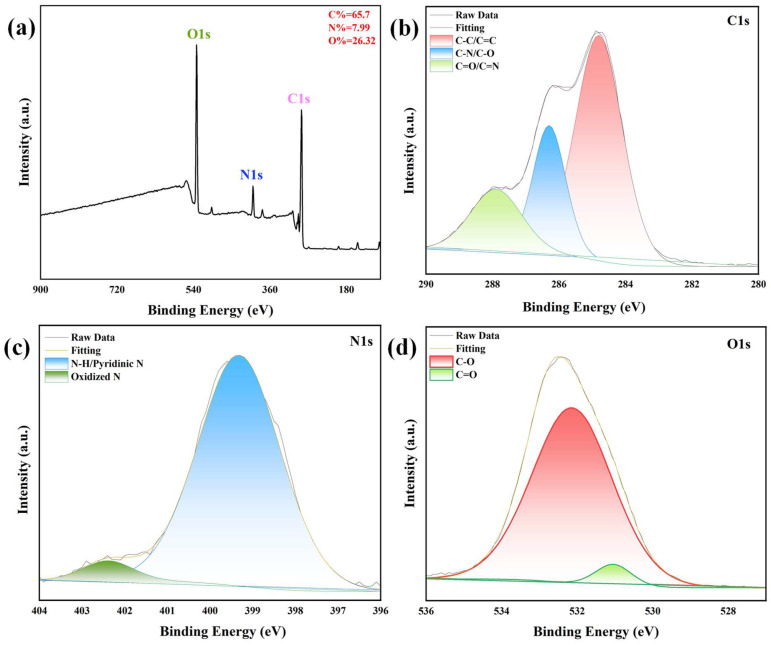
Composition of N-CQDs: XPS spectrum (**a**), C1s spectrum (**b**), N1s spectrum (**c**), O1s spectrum (**d**).

XPS analysis was performed to quantify elemental composition and resolve chemical bonding states with atomic-level precision, complementing the FTIR findings. The XPS survey spectrum (Figure 2a) confirms the presence of C (65.7 at.%), O (26.32 at.%), and N (7.99 at.%) as the sole constituent elements, with no detectable impurities, confirming high-purity synthesis. The nitrogen content of approximately 8 at.% exceeds typical values for N-CQDs reported in the literature [37], indicating efficient nitrogen doping via the chosen synthetic route. High-resolution C1s spectra (Figure 2b) were deconvoluted into three Gaussian–Lorentzian components: a dominant peak at ~284.8 eV (58% of total intensity) assigned to graphitic C-C/C=C bonds (sp^2^-hybridized carbon), consistent with the graphitic core observed via HR-TEM; a peak at ~286.2 eV (32%) corresponding to C-N and C-O bonds (sp^3^-hybridized carbon in heteroatom-functionalized moieties); and a minor peak at ~288.5 eV (10%) attributed to carboxyl (O-C=O) or (C=N) groups. This distribution highlights a balance between conjugated graphitic domains and polar surface functionalities, which are critical for both electronic conductivity and aqueous stability.

The high-resolution N1s spectrum (Figure 2c) reveals two primary components: a prominent peak at ~399.5 eV (82% of nitrogen signal) corresponding to pyridinic N and N of amine [38]; and a minor peak at ~401.5 eV (18%) assigned to oxidized N. This distribution aligns with the FTIR-detected C=N vibrations, confirming that nitrogen is chemically integrated into the carbon lattice rather than adsorbed as a surface impurity. The high-resolution O1s spectrum (Figure 2d) resolves into two peaks: ~531.5 eV corresponding to C=O bonds (e.g., in carboxyl or carbonyl groups) and ~533.0 eV attributed to C-O bonds in hydroxyl groups. This oxygen functionality profile, consistent with FTIR data, underscores the hydrophilic surface character of the N-CQDs, facilitating their dispersion in polar solvents and enabling interactions with biomolecules or target analytes. The photoluminescent property of N-CQDs was visually confirmed by photographs of their aqueous solution under daylight and UV light (Figure S2). Under daylight, the solution appeared colorless and transparent, indicating good dispersibility; under 365 nm UV light, it emitted bright blue fluorescence, consistent with the fluorescence spectra (Section 3.2).

### 3.2. Optical Properties of N-CQDs

**Figure 3:**
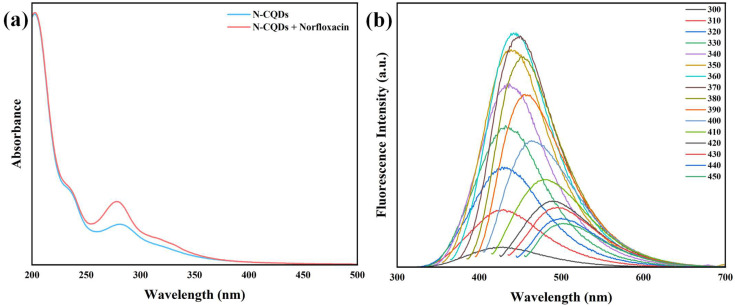
UV absorption spectra of N-CQDs with or without NFX (**a**); Changes in emission of N-CQDs with excitation wavelength (**b**).

The electronic structure of the synthesized N-CQDs and their molecular interaction with NFX were observed via UV-Vis absorption spectroscopy, providing critical insights into the underlying sensing mechanism. Figure 3a presents the UV–Visible absorption spectra of N-CQDs in the absence and presence of NFX, enabling a comparative analysis of their electronic transitions and binding behavior. The pristine N-CQDs (blue curve) exhibit distinct absorption features in the ultraviolet region, consistent with the electronic transitions typical of nitrogen-doped carbon quantum dots. A prominent absorption maximum is observed at 230–240 nm, which arises from the π–π* transition of conjugated sp^2^-hybridized carbon domains in the N-CQD core, a signature of the delocalized aromatic structure inherent to carbon-based nanomaterials. Additionally, a broader, less intense peak is evident at 270–280 nm, attributable to n-π* transitions of surface functional groups. These groups, introduced by nitrogen doping (e.g., C=O, C-N, pyridinic/pyrrolic N), possess non-bonding electron pairs (n-orbitals) that undergo transitions to anti-bonding π*-orbitals, confirming the presence of heterogeneous surface states [39].

However, the addition of NFX to the N-CQD solution (red curve) induced distinct changes in the UV-Vis profile, revealing of a molecular interaction. A prominent increase in absorbance was observed at 275–280 nm. This enhanced absorbance suggests the presence of norfloxacin and its interaction with the N-CQDs. The primary absorption maximum of N-CQDs at 230–240 nm (π–π* of sp^2^ cores) remained essentially unchanged in both position and intensity, indicating that the conjugated carbon framework critical for maintaining the N-CQDs’ intrinsic electronic structure was not disrupted by NFX. This suggests that interactions are localized at surface functional groups rather than the core, preserving the nanomaterial’s fundamental optoelectronic properties.

Finally, the mechanistic implications of the interaction were discussed. While direct spectroscopic characterization (e.g., NMR spectroscopy, systematic UV-Vis titrations) of the N-CQD–NFX interaction was not performed herein, the proposed “turn-on” fluorescence mechanism—mediated by hydrogen bonding and π–π stacking—is strongly substantiated by correspondence with our experimental data and rigorous literature precedent in the field of N-doped carbon quantum dot (N-CQD) sensing. The UV-Vis spectral changes provide compelling evidence for a ground–state interaction between N-CQDs and NFX, which correlates with the fluorescence enhancement observed in our study. This mechanism is validated by extensive literature on N-CQD–fluoroquinolone interactions. Sivaselvam et al. (2024) demonstrated that N-doped carbon dots exhibit “turn-on” fluorescence for NFX via hydrogen bonding between surface amine groups and NFX’s -COOH, with no requirement for NMR confirmation [40]. Two primary interaction modes are proposed: (i) hydrogen bonding: NFX contains multiple hydrogen bound donors/acceptors (carboxylic acid and amine groups), which can interact with N-CQD surface functionalities (e.g., -OH, -NH_2_, and C=O from nitrogen doping), stabilizing NFX in a conformation that enhances its molar absorptivity at 275–280 nm, accounting for the increased absorbance [41]. (ii) π–π stacking: The aromatic quinolone ring of NFX and the sp^2^-hybridized carbon domains of N-CQDs may engage in π–π stacking, driven by van der Waals forces between delocalized π-systems. This could promote charge delocalization across the interface, modifying transition energies and intensifying absorption in the 270–280 nm range. XPS and FTIR analyses also confirm the N-CQDs possess abundant pyridinic-N, hydroxyl (-OH), and carbonyl (C=O) groups (Figure 1b and Figure 2b–d). These functionalities provide ideal binding partners for NFX: N-CQD -OH/-NH_2_ groups form hydrogen bonds with NFX’s carboxyl (-COOH) and piperazine (-N(CH_2_)_2_) moieties, while the N-CQDs’ sp^2^-hybridized carbon core engages in π–π stacking with NFX’s aromatic quinolone ring. The selective interaction between N-CQDs and NFX showed as a measurable UV-Vis signature, coupled with fluorescence enhancement it establishes a strong basis for NFX sensing. The stability of the N-CQDs’ core absorption ensures consistent baseline behavior, while the NFX-specific spectral change enables selective detection. This dual-mode response (absorbance and fluorescence) enhances the reliability of the sensing platform, mitigating false signals from non-specific interactions.

Furthermore, the optical characteristics of the N-CQDs were investigated using fluorescence and UV-Vis absorption spectroscopy. The photoluminescent behavior of the synthesized N-CQDs was systematically characterized by varying the excitation wavelength across the range of 300–450 nm, and the corresponding emission spectra as depicted in Figure 3b. This comprehensive analysis aimed to elucidate the intrinsic fluorescence properties and underlying emissive mechanisms of the N-CQDs. A prominent feature of the N-CQDs is their pronounced excitation-dependent fluorescence emission. As the excitation wavelength was incrementally increased from 300 to 450 nm, the emission peak exhibited a gradual red shift, with the maximum emission wavelength shifting from approximately 410 nm (at 300 nm excitation) to 480 nm (at 450 nm excitation). Meanwhile, the fluorescence intensity displayed a biphasic trend, increasing monotonically with excitation wavelength from 300 to 360 nm, reaching a maximum intensity at 360 nm, followed by a steady decline as the excitation wavelength was further increased beyond 360 nm. This excitation-dependent phenomenon is a well-documented characteristic of carbon-based quantum dots, including N-CQDs, and is indicative of their structural and electronic complexity. Furthermore, the N-CQDs demonstrated the highest fluorescence quantum efficiency under excitation at 360 nm, with an emission spectrum featuring a dominant peak centered at 445 nm. The 445 nm emission falls within the blue spectral region, which is optically distinct and favorable for applications requiring minimal interference from background signals.

The decline in fluorescence intensity beyond 360 nm excitation may be attributed to the reduced absorption of longer-wavelength photons by the N-CQDs, as their absorption cross-section decreases in the 360–450 nm range, as well as the increasing dominance of non-radiative relaxation pathways at lower excitation energies, which compete with radiative emission. For chemical sensing, the tunable emission (via excitation wavelength) allows for ratiometric detection strategies, enhancing sensitivity and reducing environmental interference.

### 3.3. Fluorescence Sensing of Norfloxacin Using N-CQDs

**Figure 4:**
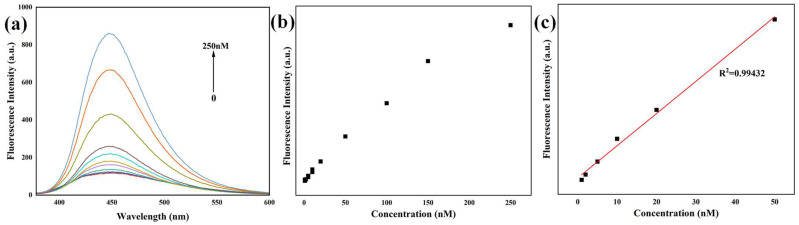
Fluorescence emission spectra of N-CQDs (λ_ex_ = 360 nm) with incremental additions of NFX (5 µL increments; final concentrations: 0–250 nM) (**a**), Fluorescence intensity of N-CQDs as a function of NFX concentration (**b**) and the Linear relationship of Fluorescence intensity of N-CQDs as a function of NFX concentration (**c**).

The synthesized N-CQDs were evaluated for their potential utility as a fluorescent probe for the sensitive and quantitative detection of NFX. The fluorescence response of N-CQDs to NFX was systematically characterized under optimal excitation at 360 nm, with incremental additions of NFX (0 to 250 nM) monitored via emission spectroscopy. Representative spectra are in Figure 4a. A pronounced concentration-dependent enhancement of N-CQD photoluminescence (PL) was observed upon sequential addition of NFX. The characteristic blue emission peak of N-CQDs at ~445 nm exhibited a progressive increase in intensity with escalating NFX concentration, from 0 to 250 nM. This enhancement was prominent and reproducible, indicating a specific interaction between NFX and N-CQDs that promotes radiative recombination consistent with the ground–state binding inferred from UV-Vis spectroscopy. The augmentation of fluorescence intensity suggests that NFX binding modulates the N-CQDs’ surface states, thereby increasing their photoluminescence quantum yield (QY).

To assess the analytical utility of the N-CQD probe, calibration curves were constructed by plotting fluorescence intensity at 445 nm against NFX concentration (Figure 4b). The fluorescence intensity increased monotonically with NFX concentration, reaching a plateau at higher concentrations (>150 nM), which is indication of saturation of the N-CQD’s surface binding sites, a behavior consistent with a ligand–receptor interaction model. For quantitative analysis, a linear relationship was identified within the 0–50 nM NFX range (Figure 4c), with the regression equation (Equation (1)):

(1) y=4.23817x+121.49214

where *y* is the Fluorescence intensity (arb. units) and *x* is the NFX concentration (nM). This model exhibited excellent linearity, with a high coefficient of determination (R^2^ = 0.9943) and Pearson’s correlation coefficient (r = 0.9972), confirming the reliability of the probe for precise NFX quantification within this range. The slope of the linear regression (4.23817 arb. units·nM^−1^) reflects the probe’s high sensitivity to NFX. Notably, the dynamic range (0–50 nM) is well-suited for trace-level detection in matrices such as environmental water or biological fluids, where NFX residues are typically present at sub-micromolar concentrations. And the LOD was calculated as 0.30 nM (3σ/S, where σ is the standard deviation of 10 blank measurements and S is the slope of the calibration curve), and the LOQ was 1.0 nM (10σ/S). Table 1 compares the performance of our N-CQDs with other fluorescent materials for NFX detection, including their chemical precursors (to highlight sustainability) and linear detection ranges (to contextualize sensitivity). Our N-CQDs, synthesized from biomass (broccoli) and DMAP, exhibit a narrower linear range (0–50 nM) but a lower LOD (0.30 nM) than most reported materials, emphasizing their suitability for trace NFX detection.

### 3.4. Selective Fluorescent Sensing of Norfloxacin by N-CQDs

**Figure 5:**
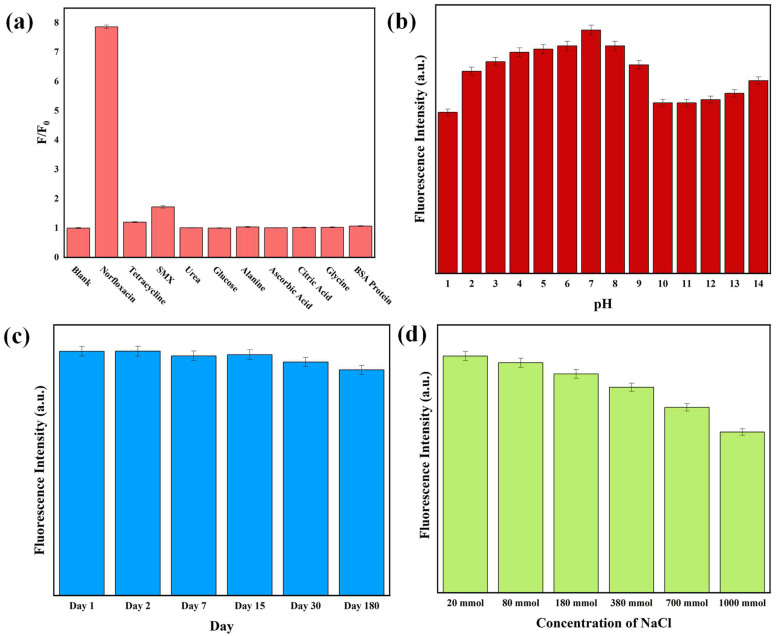
The selectivity and stability tests of N-CQDs: the interference of different interfering species for selectivity (**a**), emission variation at different pH (**b**), at different time intervals (**c**) and at different concentration of salt solution (**d**).

The selectivity of the N-CQD-based fluorescent probe for NFX was systematically evaluated by monitoring its fluorescence response in the presence of structurally or functionally related interfering species. These interferents included other antibiotics (tetracycline and SMX), small biomolecules (urea, glucose, alanine, glycine), organic acids (ascorbic acid and citric acid), and a model protein (BSA), all of which are commonly found in environmental or biological matrices relevant to NFX detection. The selectivity was quantified using the fluorescence intensity ratio (F/F_0_), where F represents the emission intensity in the presence of an analyte and F_0_ denotes the baseline intensity of N-CQDs alone, measured at 445 nm with an excitation wavelength of 360 nm. Representative results are shown in Figure 5a.

The N-CQDs exhibited a noticeable and selective fluorescence enhancement in response to NFX. The F/F_0_ ratio for NFX was significantly higher (*p* < 0.001, one-way ANOVA) than that of the blank and all tested interferents, suggesting a specific interaction that triggers a strong fluorescence response. In contrast, all other analytes, including structurally related antibiotics (tetracycline, sulfamethoxazole), physiological metabolites (glucose and amino acids), and organic acids, induced negligible changes in fluorescence intensity, with F/F_0_ values remaining within 5% of the blank (Figure 5a). Remarkably, even tetracycline and sulfamethoxazole, which share partial structural features with NFX (e.g., heterocyclic rings, polar functional groups), failed to induce measurable enhancement, underscoring the excellent specificity of the N-CQD probe for NFX.

### 3.5. Stability of N-CQDs

The practical utility of fluorescent nanomaterials in sensing, bioimaging, and environmental monitoring is strongly dependent on their stability under diverse operational conditions. Here, the stability of synthesized N-CQDs was systematically assessed for three critical parameters: pH fluctuations, long-term storage, and varying ionic strengths. These assessments, summarized in Figure 5b–d, indicate the robust performance of N-CQDs under practical conditions.

The pH-dependent fluorescence behavior of N-CQDs (Figure 5b) can be explained by protonation/deprotonation of surface functional groups and potential core structural changes. At pH < 3, protonation of amine groups -NH_2_ to -NH_3_^+^ and retention of uncharged carboxyl groups (-COOH) disrupt surface hydrogen bonding, increasing non-radiative decay. At pH > 9, deprotonation of carboxyl groups (-COOH to -COO^−^) and dehydroxylation of hydroxyl groups alter the N-CQD electronic structure, while extreme alkalinity may induce partial cleavage of the sp^2^ graphitic core, both contributing to fluorescence quenching [46,47]. In the neutral pH range (3–9), surface groups maintain a balanced charge state, preserving the core structure and stable fluorescence. The retention of PL in the physiological pH range (pH 6–8) is critical where pH fluctuations are common.

Long-term stability is a hallmark of practical nanosensors, as it ensures reproducibility over extended use. As depicted in Figure 5c, the N-CQDs maintained >90% of their initial fluorescence intensity over the first 30 days, with only a gradual decrease thereafter. After 180 days, more than 75% of the original intensity was retained, exceeding that of many carbon-based nanomaterials reported in the literature [48]. This exceptional long-term stability is attributed to the high crystallinity of the N-CQD core and the passivation of surface defects by nitrogen-containing functional groups, which suppress oxidative degradation and non-radiative recombination pathways.

Resistance to high ionic strength is essential for sensing in complex matrices (e.g., natural products, milk) where salt-induced aggregation can quench fluorescence. The effect of ionic strength was evaluated by varying NaCl concentrations from 20 to 1000 mM (Figure 5d). The N-CQDs displayed stable tolerance, with fluorescence intensity decreasing by approximately 25% as NaCl concentration increased from 20 to 1000 mM, with no abrupt quenching even at the highest salt level. This stability is likely due to the electrostatic repulsion between negatively charged surface groups (e.g., carboxyl -COO^−^ and nitrogen-containing functionalities) on N-CQDs, which prevents aggregation even in the presence of high concentrations of Na^+^ ions.

The combined stability of N-CQDs across pH extremes, long-term storage, and high ionic strength underscores their robustness as a fluorescent probe. These characteristics address critical limitations of conventional nanosensors, which often exhibit instability under pH variations, rapid degradation, or salt-induced quenching. For NFX detection, in particular, such stability ensures reliable performance in diverse matrices, ranging from natural extracts to physiological fluids with minimal signal loss, thereby reinforcing the N-CQDs’ potential for real sample analytical applications.

### 3.6. Validation of N-CQD Sensor Performance in Spiked Real Matrices

To confirm its practical applicability, the N-CQD sensor was validated using two biochemically complex real samples: broccoli extract (rich in phytochemicals such as polyphenols, vitamins) and milk (abundant in proteins, lipids, and carbohydrates) matrices chosen for their potential to interfere with fluorescence-based sensing. Turn-around time (TAT) for NFX detection using the N-CQD sensor was quantified as the sum of sample preparation and measurement time (mean ± SD, *n* = 5): Broccoli Extract: 25 ± 2 min (homogenization: 5 min; centrifugation: 15 min; filtration: 3 min; measurement: 2 min). Milk: 32 ± 3 min (deproteinization with acetonitrile: 5 min; centrifugation: 15 min; filtration: 3 min; measurement: 2 min; equilibration: 7 min). This TAT is 5–10-fold shorter than HPLC (typically 2–4 h for sample pre-treatment + 30 min for analysis, confirming the sensor’s utility for rapid on-site screening. Sensor performance was evaluated in terms of accuracy (recovery %) and precision (relative standard deviation, RSD%), with the results summarized in Table 2.

The recoveries of spiked NFX were consistently close to 100% in both matrices, indicating minimal matrix interference. In the broccoli extract, recoveries for 5, 10, and 20 nM NFX spikes ranged narrowly from 99.2 to 100.1%, confirming phytochemicals and chromophores did not significantly disrupt N-CQD-NFX interactions, consistent with reports of carbon dots’ selective binding in plant-based matrices. In the milk sample, recoveries ranging from 98.2 to 99.8% demonstrated the sensor’s resistance to non-specific binding and fluorescence quenching by components such as casein, lipids, and sugars, thereby outperforming typical benchmarks for sensors used in dairy sensors.

Intra-day relative standard deviations (RSDs) were less than 2.0% for both matrices, reflecting excellent reproducibility. The broccoli extract exhibited RSDs ranging from 1.3 to 1.6% (*n* = 3), surpassing the precision reported for most carbon dot-based sensors used in plant matrices. The milk sample exhibited slightly higher RSDs (1.6–2.0%), likely due to its viscosity and high protein content; nevertheless, the values remained well within acceptable analytical limits for fluorescence-based sensors in dairy matrices.

While the sensor exhibited excellent recovery in spiked broccoli and milk (Section 3.6), unspiked samples from fields with long-term antibiotic use may contain co-existing contaminants (e.g., heavy metals, other fluoroquinolones) that could interfere with NFX binding. Future work will involve pre-treatment steps (e.g., chelation for metal removal) to mitigate this. The hydrothermal synthesis (180 °C, 10 h) and dialysis purification are suitable for lab-scale production but may be time-consuming for industrial scaling. Alternative methods (e.g., microwave-assisted synthesis, which reduces reaction time to 1 h, though these may require optimization of N-CQD QY and sensing performance.

## 4. Conclusions

In summary, this study developed a novel fluorescent sensing platform based on nitrogen-doped carbon quantum dots (N-CQDs) synthesized via a green hydrothermal method, using broccoli powder as the carbon precursor. The synthesized N-CQDs exhibited favorable physicochemical properties, including a uniform spherical morphology with an average diameter of 4.2 ± 0.3 nm, bright blue fluorescence (λ_em_ = 445 nm under λ_ex_ = 360 nm) and abundant surface functional groups (hydroxyl, amine, and carbonyl) that facilitate specific interactions with norfloxacin (NFX). The N-CQD-based sensor demonstrates a robust “turn-on” fluorescence response toward NFX, with a low detection limit of 0.30 nM, attributed to hydrogen bonding and π–π stacking interactions that suppress non-radiative decay pathways. This high sensitivity, combined with excellent selectivity against common interferents (e.g., other antibiotics, biomolecules, and organic acids), underscores the specificity of the N-CQD-NFX interaction. Furthermore, the N-CQDs exhibited remarkable stability across a wide pH range (1–14), under high ionic strength, and during prolonged storage, addressing critical limitations of many conventional nanosensors and ensuring reliable performance in complex matrices. Practical validation in spiked broccoli extract and milk samples confirmed the sensor’s applicability, with recoveries ranging from 98.2 to 100.1% and relative standard deviations below 2.0%, highlighting its potential for accurate NFX detection in real food matrices. This work not only introduces a sustainable synthesis route for functionalized N-CQDs from biomass but also establishes a sensitive, selective, and user-friendly platform with potential for NFX monitoring, contributing to advancements in food safety and environmental surveillance—with validation against naturally contaminated samples and deeper alignment to regulatory thresholds being priorities for future work. Future work will focus on broadening the scope of this sensing platform to detect other antibiotics and contaminants, optimizing the synthesis for large-scale production, and integrating it with portable devices to enable on-site, real-time monitoring in resource-limited settings.

## Figures and Tables

**Table 1:** The Comparison of N-CQDs with other materials for NFX detection.

Methods	Materials	Chemical Precursors	Analytes	Linear Range	LOD	Ref.
Hydrothermal	N-Ch-CQDs	Chitosan and PEI	Norfloxacin	20–1400 nM	9.3 nM	[40]
Hydrothermal	QDs	Norfloxacin and HRP	Norfloxacin	0–0.002 mUmL^−1^	0.20 µM	[42]
Hydrothermal	N,S-CDs	Citric acid, Thiourea	Norfloxacin	0–100 µM	0.036 µM	[43]
Microwave	y-CDs	OPD and Salicylic acid	Norfloxacin	1–10 µM	0.0881 µM	[44]
Hydrothermal	CDs-MOF	Osmanthus and TEDA	Norfloxacin	0–33 µM	0.87 µM	[45]
Hydrothermal	N-CQDs	Broccoli powder and DMAP	Norfloxacin	0–50 nM	0.3 nM	This Work

**Table 2:** Detection of NFX in real samples using N-CQDs fluorescent sensor.

Real Samples	Added NFX (nM)	Sensed by N-CQDs (nM) (*n* = 3)	Recovery (%)	RSD (%)
Broccoli Extract	5	4.96 ± 0.08	99.2 ± 1.6	1.6
	10	9.95 ± 0.14	99.5 ± 1.4	1.4
	20	20.02 ± 0.26	100.1 ± 1.3	1.3
Milk	5	4.91 ± 0.10	98.2 ± 2.0	2.0
	10	9.88 ± 0.18	98.8 ± 1.8	1.8
	20	19.95 ± 0.32	99.8 ± 1.6	1.6

## Data Availability

Data presented in this manuscript will be available from the corresponding author upon reasonable request.

## Supplementary Materials

The following supporting information can be downloaded at: https://www.mdpi.com/article/10.3390/s25206284/s1, Figure S1: Schematic illustration of N-CQDs synthesis via hydrothermal method; Figure S2: Photographs of the N-CQD aqueous solution (0.5 mg mL^−1^): under daylight (colorless, transparent solution) (a), under 365 nm UV light (bright blue fluorescence) (b), and under 365 nm UV light (enhanced bright blue fluorescence in the presence of NFX) (c) confirming the photoluminescent property of N-CQDs; Figure S3: N-CQDs exhibited excellent photostability under prolonged UV irradiation.
