# Supplementary material for: Broccoli to the Lab: Green-Synthesized N-CQDs for Ultrasensitive “Turn-On” Detection of Norfloxacin in Food
Source: Sensors (Basel). 2025 Oct 10;25(20):6284. doi: 10.3390/s25206284 (PMC12567167; doi:10.3390/s25206284)
Supplement: Supplementary file 1 [file sensors-25-06284-s001.zip › sensors-3895763-supplementary.pdf]

Supplementary Materials

Broccoli to the Lab: Green-Synthesized N-CQDs for Ultrasensitive "Turn-On" Detection of Norfloxacin in Food

Zubair Akram [1], Anam Arshad [1], Sajida Noureen [2], Muhammad Mehdi [3], Ali Raza [1], Nan Wang [1,4,*] and Feng Yu [1,4,*]

[1] Key Laboratory for Green Processing of Chemical Engineering of Xinjiang Bingtuan, School of Chemistry and Chemical Engineering, Shihezi University, Shihezi 832003, China; zubair.a.sidhu@gmail.com (Z.A.); anamarshad9293@gmail.com (A.A.); razaali2021@stu.shzu.edu.cn (A.R.)
[2] Materials Chemistry Laboratory, Institute of Chemistry, The Islamia University of Bahawalpur, Baghdad-ul-Jadeed Campus, Bahawalpur 63100, Pakistan; sajida.noureen@iub.edu.pk
[3] College of Chemistry & Pharmacy, Northwest A&F University, Yangling 712100, China; kh.mehdiali@nwafu.edu.cn
[4] Carbon Neutralization and Environmental Catalytic Technology Laboratory, Bingtuan Industrial Technology Research Institute, Shihezi University, Shihezi 832003, China
* Correspondence: nanwangsearl@outlook.com (N.W.); yufeng05@mail.ipc.ac.cn (F.Y.)

Comment: Provide a schematic illustration of the synthesis of N-CQDs.

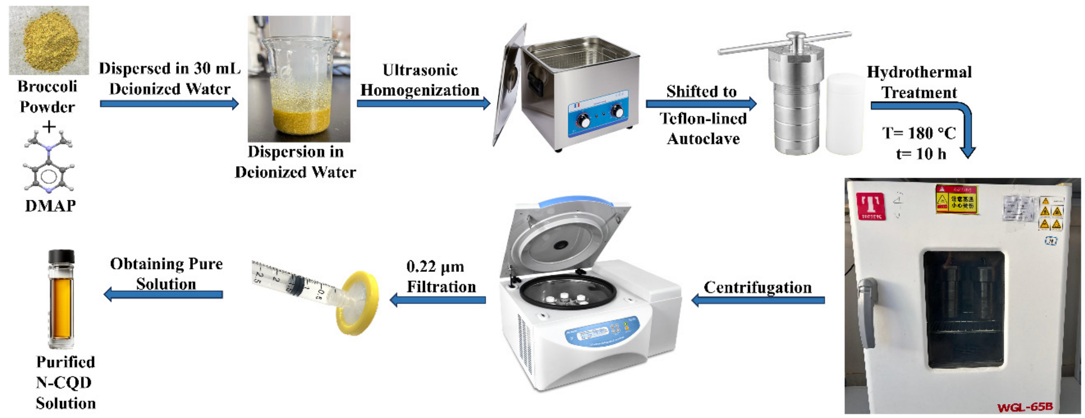

Figure S1. Schematic Illustration of N-CQDs Synthesis via Hydrothermal Method.

This schematic visually depicts the key steps: (1) dispersion of broccoli powder (carbon precursor) and DMAP (nitrogen dopant/functionalizing agent) in deionized water; (2) ultrasonic homogenization; (3) hydrothermal treatment at 180 °C for 10 h in a Teflon-lined autoclave; (4) post-synthesis purification (centrifugation, 0.22 µm filtration, and dialysis with 1000 Da MWCO membrane); and (5) final collection of purified N-CQDs as aqueous solution.

Comment: The photographs of N-CQDs in daylight and under UV light should be presented.

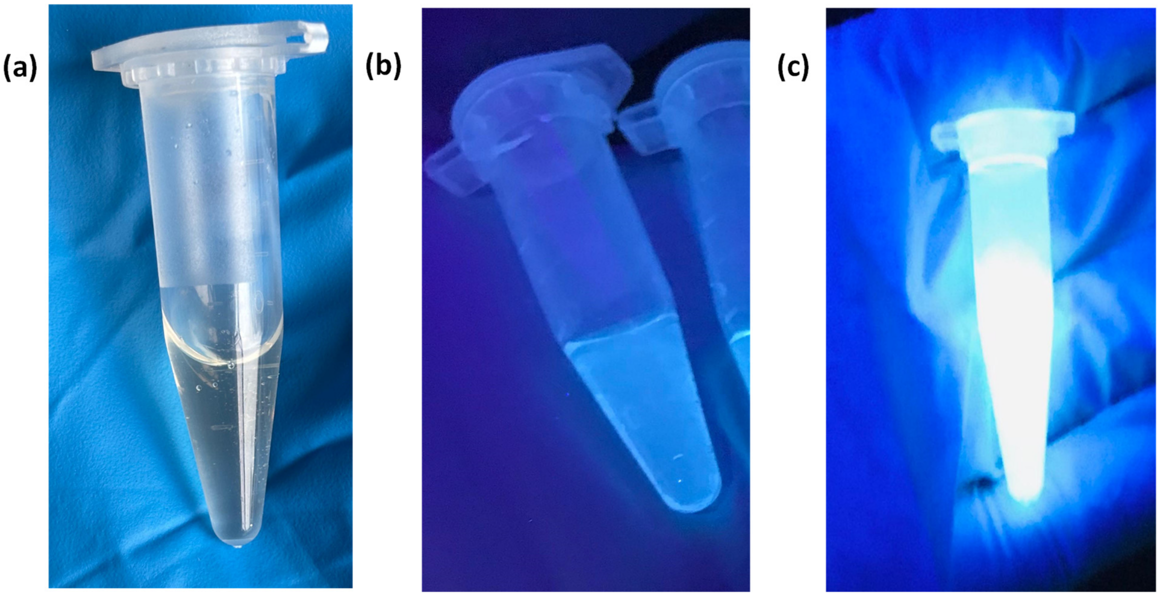

Figure S2. Photographs of the N-CQD aqueous solution (0.5 mg mL^{-1}): under daylight (colorless, transparent solution) (a), under 365 nm UV light (bright blue fluorescence) (b), and under 365 nm UV light (enhanced bright blue fluorescence in the presence of NFX) (c) confirming the photoluminescent property of N-CQDs.

The photoluminescent property of N-CQDs was visually confirmed by photographs of their aqueous solution under daylight and UV light (Figure S2). Under daylight, the solution appeared colorless and transparent, indicating good dispersibility; under 365 nm UV light, it emitted bright blue fluorescence, consistent with the fluorescence spectra (Section 3.2) and under 365 nm UV light in the presence of NFX, it emitted enhanced bright blue fluorescence, consistent with the fluorescence spectra (Section 3.3) Fluorescence Sensing of Norfloxacin Using N-CQDs.

Was the photostability of the N-CQDs solution checked under UV light? These details are described in section 2.6, but they are not found anywhere in the manuscript. Also, add the corresponding fluorescence images.

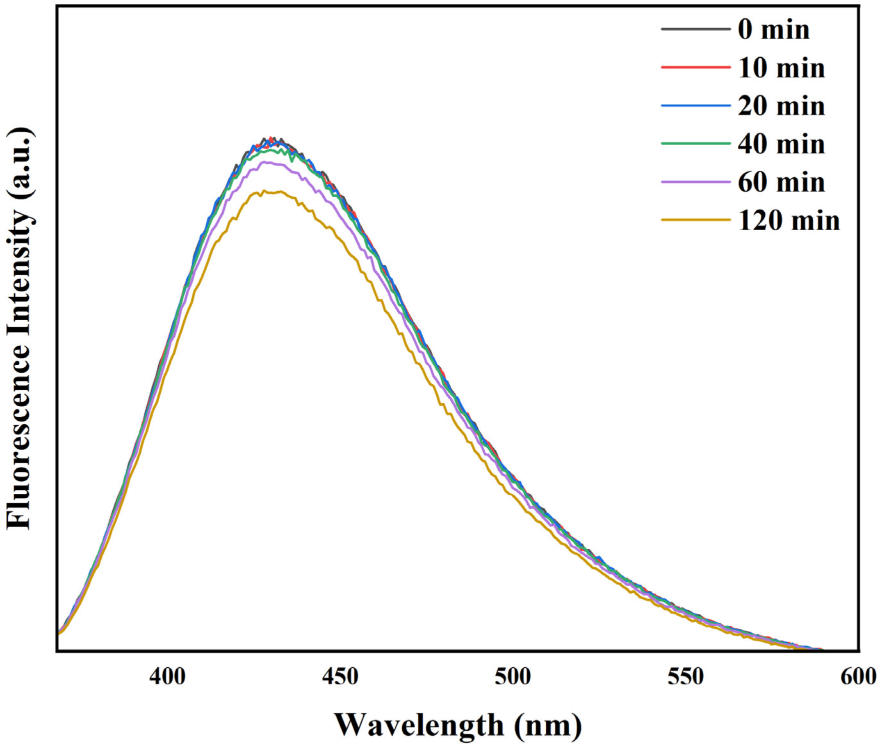

Figure S3. N-CQDs exhibited excellent photostability under prolonged UV irradiation.

The N-CQDs also exhibited excellent photostability under prolonged UV irradiation (365 nm, 10 W): after 120 mins, they retained 92% of initial fluorescence intensity (Figure S3). This ensures reliable signal acquisition during fluorescence measurements.a
